# Crocin from saffron ameliorates allergic airway inflammation through NF-κB, IL-17, and Nrf2/HO-1 signaling pathways in mice 

**DOI:** 10.22038/ijbms.2024.80614.17447

**Published:** 2024

**Authors:** Farhad Jeddi, Sara Zahertar, Ali Bordbar, Ramin Salimnejad, Hassan Ghobadi, Mohammad Reza Aslani

**Affiliations:** 1 Department of Genetics and Pathology, School of Medicine, Ardabil University of Medical Sciences, Ardabil, Iran; 2 Lung Diseases Research Center, Faculty of Pharmacy, Ardabil University of Medical Sciences, Ardabil, Iran; 3 Department of Anatomical Sciences, School of Medicine, Ardabil University of Medical Sciences, Ardabil, Iran; 4 Lung Diseases Research Center, Ardabil University of Medical Sciences, Ardabil, Iran; 5 Applied Biomedical Research Center, Mashhad University of Medical Sciences, Mashhad, Iran

**Keywords:** Asthma, Crocin, Heme oxygenase-1, Nuclear erythroid 2-related - factor 2, Ovalbumin

## Abstract

**Objective(s):**

Asthma is a complex inflammatory disorder with the infiltration of inflammatory cells in the lung airways. Saffron’s active component, crocin, has been proven to possess anti-inflammatory and anti-oxidant effects. The objective of this current study was to explore the impact of crocin on NF-kB and nuclear erythroid 2-related factor 2 (Nrf2)/ heme oxygenase-1 (HO-1) signaling pathways in ovalbumin (OVA)-sensitized mice, aiming to understand its mechanism.

**Materials and Methods:**

Four different groups were formed by dividing forty male BALB/C mice: control group, OVA-sensitized group (OVA), OVA combined with crocin 30 mg/kg (OVA-Cr30), and the OVA combined with crocin 60 mg/kg (OVA-Cr60). In order to determine the total number of WBC and inflammatory cells infiltrating the lung, we utilized the bronchoalveolar lavage fluid for counting purposes. The mRNA and protein levels of Nrf2, HO-1, IL-17, and NF-κB in lung tissue were assessed through real-time PCR and western blot techniques.

**Results:**

Crocin significantly prevented the increase of total WBC and inflammatory cells in the lung tissue (*P*<0.001 for all) and histopathological changes in OVA-sensitized mice. Furthermore, crocin displayed suppressive effects on the enhancement of NF-kB (*P*<0.01) and IL-17 (*P*<0.05) mRNA and protein levels in OVA-sensitized mice while preserving Nrf2 (*P*<0.01) and HO-1 (*P*<0.05) expression levels. Crocin effects became increasingly apparent when utilized at high concentrations.

**Conclusion:**

Crocin decreased airway inflammation, partially by inhibiting NF-κB and IL-17 and up-regulating Nrf2/HO-1 mRNA and protein expression levels.

## Introduction

Bronchial asthma causes an ongoing inflammation in the airways marked by the infiltration of inflammatory cells such as neutrophils, eosinophils, and lymphocytes into the lung tissue (1). Furthermore, asthma has more prominent features, including airway hyperresponsiveness (AHR), mucus hypersecretion, and lung tissue remodeling (2). The pathogenesis of asthma has been linked to an imbalance between Th2 and Th1 cells, resulting in increased levels of interleukin (IL)-4, IL-5, and IL-13 and decreased levels of interferon (IFN)-γ (3). Also, the Th2/Th1 imbalance results in eosinophilia and the release of immunoglobulin (Ig) E into the lung (3). It has also been reported that in severe and treatment-resistant asthma, Th17 cell activation leads to the release of inflammatory mediators IL-17A and IL-17F (4). Moreover, findings from animal studies suggest that the coexistence of obesity and asthma triggers the activation of Th1, Th2, and Th17 cells, which could be responsible for the exacerbation of asthma symptoms (4-6). 

Asthmas pathophysiology involves multiple signaling pathways, with *nuclear factor kappa B* (NF-kB) as an essential transcription factor mediating inflammatory and immune responses (7, 8). Animal studies have demonstrated that inhibiting NF-κB, a pathway activated in asthma, promotes the decreased production of inflammatory cytokines, ultimately leading to reduced asthma symptoms (9). Among the noteworthy signaling pathways in asthma research, the nuclear factor-erythroid 2-related factor 2 (Nrf2) and heme oxygenase-1 (HO-1) have attracted considerable attention. Through its association with anti-oxidant-response element (ARE) genes, Nrf2 exerts anti-inflammatory and anti-oxidant effects (10). It has been documented that activating the Nrf2/HO-1 pathway in asthma leads to detectable decreases in AHR, mucus hypersecretion, and Th2 cytokine release (11). Therefore, suppressing the NF-kB pathway and potentiating the Nrf2/HO-1 pathway have been considered therapeutic targets in patients with asthma.

Recently, studies have emphasized plant-derived and naturally-occurring Nrf2 activators (12-14). Human and animal studies have revealed the potential therapeutic effects of saffron and its active ingredients (crocin, crocetin, safranal, and picrocrocin) in treating different inflammatory disorders such as asthma, *chronic obstructive pulmonary disease* (COPD), obesity, diabetes, cancer, metabolic syndrome, polycystic ovary syndrome (PCOS), and cardiovascular disease (15-19). Anti-inflammatory, anti-oxidant, and anti-cancer effects make crocin a highly significant substance. In some studies, activating the Nrf2/HO-1 pathway is believed to be responsible for crocin anti-oxidant and anti-inflammatory effects (20, 21). Despite numerous studies on the beneficial effects of crocin in treating asthma in animal models, limited focus has been placed on investigating the potential involvement of the Nrf2/HO-1 pathway. To address this possibility, this study aimed to explore the impacts of crocin on NF-kB, IL-17, and Nrf2/HO-1 pathways in a murine model of asthma.

***Experimental procedures***

*Animals and studied groups*

Forty male BALB/C mice weighing 20-25 g were included in the current research. Animals sourced from Tehran Pasteur Institute underwent a one-week acclimatization at Ardabil University of Medical Sciences animal house. In order to conduct the study, animals were given unlimited access to food and water, and their living conditions consisted of standard settings, including a regulated temperature of 22±2 ^ο^C and an equal distribution of dark/light periods spanning 12 hr each.

Four groups were created, with ten animals in each group, including the control group, the OVA-sensitized group (OVA), the OVA-sensitized combined with crocin 30 mg/kg group (OVA-Cr30), and the OVA-sensitized combined with crocin 60 mg/kg group (OVA-Cr60). The doses and administration forms of crocin were derived from previous reports (22). Crocin (purity assay of ≥ 98%) was purchased from Sigma (Sigma, 17204). 

*Animals ovalbumin sensitization*

**Figure 1:**
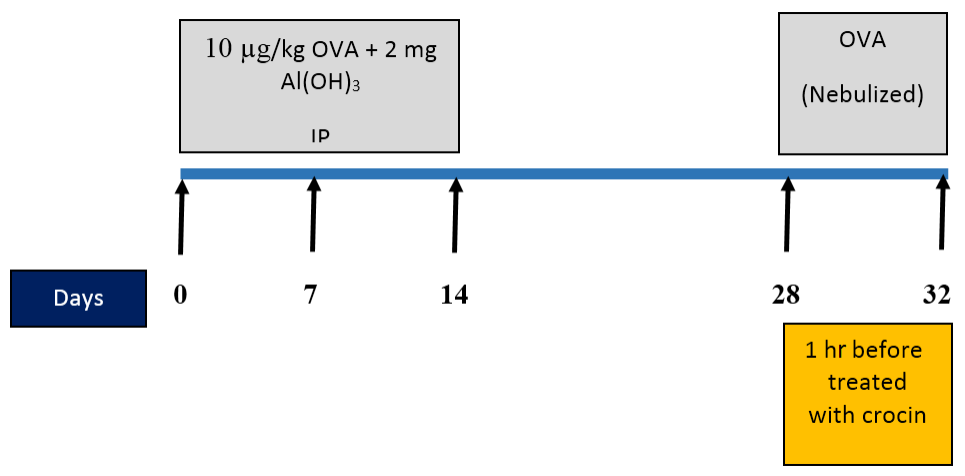
Experimental design flow chart and treatment with saline and crocin in male BALB/C mice (last 5 days of the model)

OVA: Ovalbumin; IP: Intraperitoneally

On days 0, 7, and 14, a solution of ovalbumin (10 µg) with Al (OH)_3_ (2 mg) was injected intraperitoneally to sensitize with OVA. Once the animals reached the 28th day, they were placed within a closed chamber (50 cm × 35 cm × 35 cm in size) and exposed to an aerosol of OVA at a concentration of 1% for 25 min using a nebulizer (CX3, Omron Health Care Europe B.V., the Netherlands) (23, 24). In the intervention groups, an intraperitoneal injection of crocin with a predetermined concentration was administered one hour before the ovalbumin challenge. Instead of using OVA, only saline was utilized for the control group during the same procedure (Figure 1). Animal handling was approved by the Ethical Committee of Ardabil University of Medical Sciences (IR.ARUMS.REC.1400.078).

*Determination of total WBC and differential inflammatory cells in BALF*

Following the completion of the study, the animals were given ketamine (80 mg/kg, IP) and xylazine (10 mg/kg, IP) for anesthesia and then underwent tracheal cannulation using a catheter. The process of collecting the bronchoalveolar lavage fluid (BALF) involved injecting and aspirating 0.5 ml of phosphate buffer saline (PBS) into the lung (3 to 5 times), followed by the extraction of 1.5 to 2 ml of liquid. The centrifuged BALF sample (for 10 min at 4^ο^ C at 2500 rpm) and the prepared supernatant were used for total white blood cell (WBC) and differential cell counting (25).

Total WBCs were counted using a hemocytometer and Wright-Giemsa staining technique, whereas differential cell counts followed a standard procedure of examining 200 cells per slide (×400 light microscope).

*Real-time polymerase chain reaction*

As previously reviewed in detail (26), we employed the real-time PCR methodology to measure the expression of Nrf2, HO-1, IL-17, and NF-kB mRNA levels. Table 1 displays the sequences of forward and reverse primer sets. The PCR products were normalized using the GAPDH gene, and the fold changes of gene expressions were calculated by the ΔΔCT method.

*Tissue sampling and ELISA and western blot assay*

Following the administration of anesthesia and euthanizing the animals, the lung tissue was isolated and promptly frozen in liquid nitrogen. It was then transferred to -70 ^ο^C until the expression of NF-kB, HO-1, and Nrf2 proteins were measured. 

After homogenizing lung tissue in PBS (pH 7.2-7.4), the OVA-specific IgE protein level was determined by centrifugation at 4 °C with 3000 rpm for 20 min. The supernatant was prepared using a mouse ELISA kit (according to Crystal Day’s manufacturer’s instructions).

For western blot assay, in order to create tissue homogenate, RIPA lysate was introduced into the samples while immersing them in an ice bath. To clarify the lysates, the samples underwent centrifugation at 13,300 g for 20 min at 4 °C. Protein concentration of the supernatant was measured using the BCA Protein Kit. Equal amounts of protein were separated using gel electrophoresis with sodium dodecyl lauryl sulfate. Afterward, the proteins were transferred onto membranes made of polyvinyl difluoride and then incubated with a 5% non-fat dry milk solution for two hours at room temperature in order to inhibit non-specific binding sites (27).

Primary antibodies against Nrf2 (SANTA CRUZ: sc-365949; 1:100), HO-1 (Abcam; ab13243; 1:2000), NF-kB (Abcam; ab16502; 1:1000), and β-actin (SANTA CRUZ: sc-47778; 1:1000) were added and incubated overnight at 4 °C. An advanced chemiluminescence (ECL) detection reagent was used to develop electrochemiluminescence.

*Histopathological assessment*

Once taken out, the lungs underwent fixation in 10% neutral buffered formalin and were subsequently embedded in paraffin blocks. Examination under a light microscope was conducted after cutting the tissue to 5-μm thickness and staining it with hematoxylin-eosin. The pathological criteria under investigation include epithelial destruction of airways, pulmonary fibrosis, lymphocyte cell infiltration, and hyperemia. Evaluating lung histopathological changes involved the following scoring process: 0 = normal; 1 = patchy injury, 2 = local injuries, and 3 = scattered injuries (28).

***Statistical analysis***

The results were reported as a mean (± SD). ANOVA test and Tukey-Kramer *post hoc* test were utilized to compare groups. The test significance criterion was *P*<0.05. GraphPad Prism 7 was used to draw the graph, and statistical tests were performed using SPSS (version 21).

## Results

***Total WBC count in BALF***

A notable increase in total WBCs occurred in OVA-sensitized mice after sensitization with ovalbumin (*P*<0.001). Both concentrations of crocin (30 and 60 mg/kg) displayed significant efficacy in preventing the rise in total WBC count, markedly with high concentrations of crocin (for both *P*<0.001) (Table 2). 

***Comparison of differential inflammatory cells in BALF***

Analysis of cell counts demonstrated a significant increase in the number of eosinophil, neutrophil, macrophage, and lymphocyte cells within the OVA-sensitized group compared to the control group. Both doses of crocin (30 and 60 mg/kg) exhibited significant efficacy in preventing the elevated levels of all differential inflammatory cells, with a stronger impact noted at the concentration of 60 mg/kg (Table 2).

***Effects of crocin on OVA-specific IgE levels***

**Figure 2:**
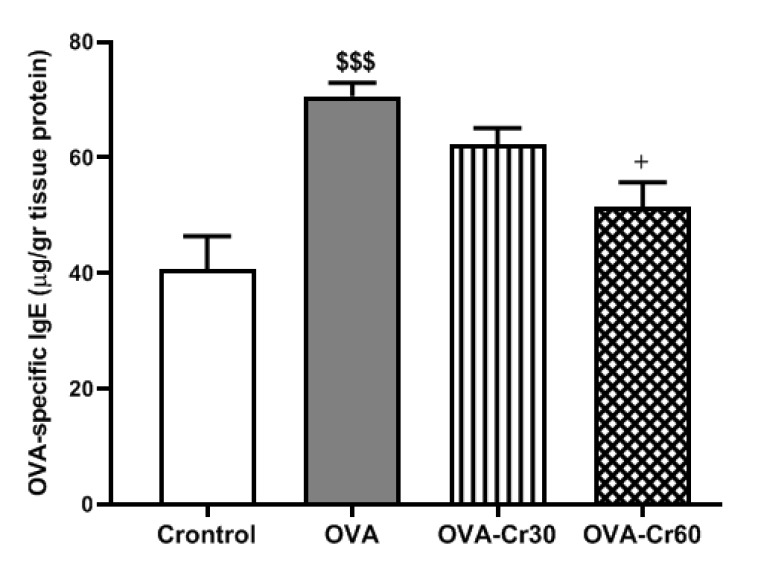
The protein level of OVA-specific IgE in lung tissue of male BALB/C mice

In contrast to the control group, the lung tissue of the OVA-sensitized mice showed significantly high levels of OVA-specific IgE (*P*<0.001). Treatment with 60 mg/kg crocin significantly decreased the serum levels of OVA-specific IgE compared to the OVA group (*P*<0.05). There was no significant difference at the 30 mg/kg crocin concentration between the OVA and OVA-Cr 60 groups (Figure 2). 

***Effects of crocin on Nrf2 and HO-1 mRNA levels***

**Figure 3:**
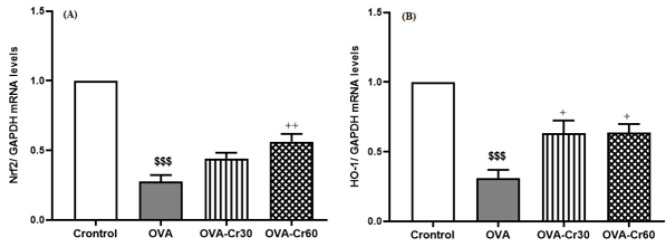
Gene expression levels of (A) Nrf2 and (B) HO-1 in lung tissue of male BALB/C mice

Nrf2 and HO-1 expression levels were found to be significantly lower in the OVA-sensitized group as opposed to the control group (for both *P*<0.001). Crocin at 60 mg/kg significantly prevented the decrease in the expression of Nrf2 (*P*<0.01) and HO-1 (*P*<0.05) in the lung tissue of OVA-sensitized mice (Figure 3).

***Effects of crocin on IL-17 and NF-kB gene expression levels***

**Figure 4:**
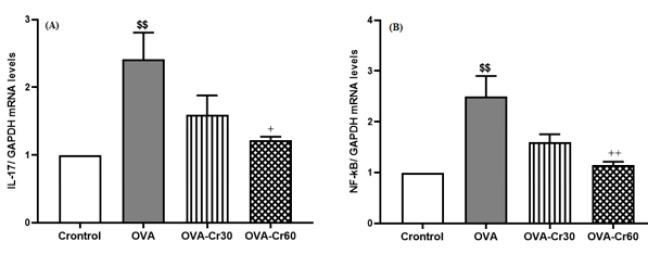
Gene expression levels of (A) IL-17 and (B) NF-kB in lung tissue of male BALB/C mice

Compared to the control group, there was a significant increase in the expression of IL-17 and NF-kB mRNA levels in the lung tissue of OVA-sensitized mice (for both *P*<0.01). Intervention with crocin 60 mg/kg significantly prevented the increase of IL-17 (*P*<0.05) and NF-kB (*P*<0.01) expression levels, while there was no significant difference between the concentrations of 30 mg/kg and 60 mg/kg crocin (Figure 4).

***Effects of crocin on Nrf2, HO-1, and NF-kB protein expression levels***

Figure 3 shows the western blot results. Significantly lower expression levels of Nrf2 and HO-1 proteins were observed in the OVA-sensitized animals (for both *P*<0.001). By utilizing crocin at concentrations of 30 (*P*<0.05) and 60 mg/kg (*P*<0.001), the reduction in Nrf2 protein expression was significantly suppressed. Regarding HO-1 protein expression, only the concentration of 60 mg/kg of crocin showed inhibitory effects related to its reduction (*P*<0.001).

**Figure 5:**
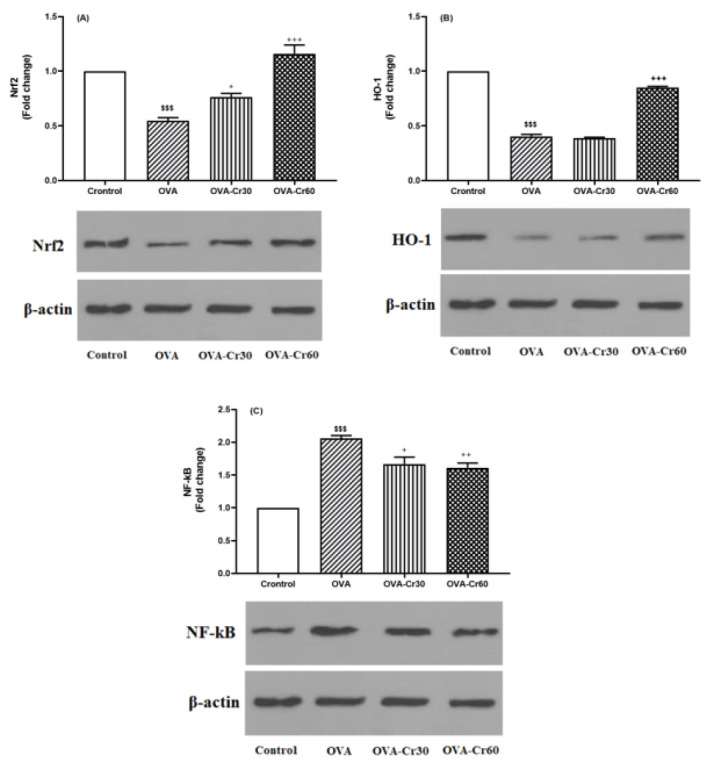
Protein expression levels of (A) Nrf2, (B) HO-1, and (C) NF-kB in lung tissue of male BALB/C mice

In the lung tissue of OVA-sensitized mice, there was a significant elevation in NF-kB protein expression levels compared to the control group (*P*<0.001). Intervention with both concentrations of 30 and 60 mg/kg of crocin significantly prevented the increase in NF-kB protein expression in the lung tissue of OVA-sensitized groups (*P*<0.05 and *P*<0.01, respectively) (Figure 5).

***Lung histopathology***

**Figure 6:**
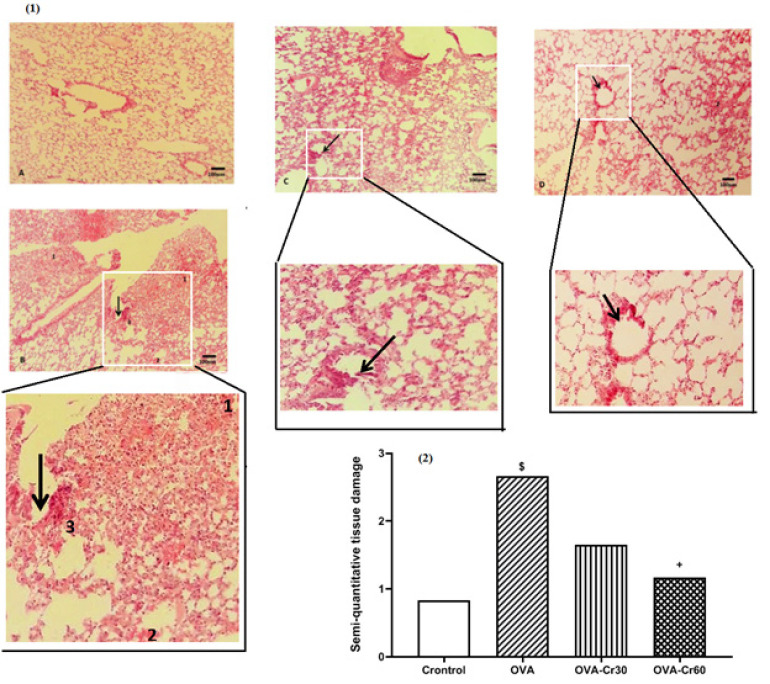
Histopathological evaluation in (1) lung tissue and (2) tissue damage index in male BALB/C mice

Ovalbumin sensitization resulted in histopathological changes characterized by the destruction of airway epithelium, lung fibrosis, hyperemia, and lymphocyte cell infiltration. By employing a semi-quantitative approach, it was determined that the utilization of crocin led to a notable reduction in tissue damage, with the most prominent effects observed at a concentration of 60 mg/kg (Figure 6).

**Table 1:** Sequences of forward and reverse primer sets in mice

Gene	Forward	Reverse
Nrf2	GCCCACATTCCCAAACAAGA	TCTCTGCCAAAAGCTGCATAC
HO-1	ATGCCCCAGGATTTGTCTGA	AGCATTCTCGGCTTGGATGT
IL-17	AGCAGCGATCATCCCTCAAA	GAAGTCCTTGGCCTCAGTGT
NF-kB	GGGAAGGATTTGGGGACTTT	CCTCCGAAGCTGAACAAACAC
GAPDH	ATGGTGAAGGTCGGTGTGAA	GAGGTCAATGAAGGGGTCGT

Nrf2: Nuclear erythroid 2-related factor 2; HO: Heme oxygenase-1; IL: Interleukin; NF-kB: Nuclear factor kappa B; GAPDH: Glyceraldehyde 3-phosphate dehydrogenase

**Table 2:** Bronchoalveolar lavage fluid cellularity in male BALB/C mice

	Control	OVA	OVA-Cr30	OVA-Cr60
WBC (× 10^5^/BAL)	2.22 ± 0.19	13.62 ± 1.28***	9.30 ± 0.52+++	6.66 ± 0.42+++, &&&
Eosinophil (× 10^5^/BAL)	0.30 ± 0.02	7.97 ± 0.72***	5.05 ± 0.24+++	3.05 ± 0.19+++, &&&
Neutrophils (× 10^5^/BAL)	0.30 ± 0.02	1.06 ± 0.17***	0.90 ± 0.08+++	0.72 ± 0.06+++
Lymphocyte (× 10^5^/BAL)	0.40 ± 0.03	1.36 ± 0.07***	0.92 ± 0.09+++	0.83 ± 0.05+++
Macrophage (× 10^5^/BAL)	1.20 ± 0.12	3.18 ± 0.35***	2.40 ± 0.19+++	2.04 ± 0.20+++

Total and differential cellularity in bronchoalveolar lavage fluid. Values are represented as mean (± SD). Statistical differences between the Control and OVA group, *** *P*<0.001. Statistical differences between OVA and other groups, +++ *P*<0.001. Statistical differences between OVA-Cr30 and OVA-Cr60 groups, &&&: *P*<0.001.

OVA: Ovalbumin, Cr: Crocin, BAL: Bronchoalveolar lavage, WBC: White blood cell

Data are expressed as mean ± SD. OVA: Ovalbumin, Cr: Crocin. Statistical differences between the Control and OVA group, $$$: *P*<0.001. Statistical difference between OVA vs other groups: +: *P*<0.05. Use the ANOVA test to compare between groups.

Data are expressed as mean ± SD. OVA: Ovalbumin, Cr: Crocin, Nrf2: Nuclear factor erythroid 2-related factor 2, and HO-1: *Heme oxygenase 1*. Statistical differences between the Control and OVA group, $$$: *P*<0.001. Statistical difference between OVA vs other groups: +: *P*<0.05, ++: *P*<0.01. Use the ANOVA test to compare between groups.

Data are expressed as mean ± SD. OVA: Ovalbumin, Cr: Crocin, IL: Interleukin, and NF-kB: Nuclear factor kappa B. Statistical differences between the Control and OVA group, $$: *P*<0.01. Statistical difference between OVA vs other groups: +: *P*<0.05, ++: *P*<0.01. Use the ANOVA test to compare between groups.

Data are expressed as mean ± SD. OVA: Ovalbumin, Cr: Crocin, Nrf2: Nuclear factor erythroid 2-related factor 2, HO-1: Heme oxygenase 1, and NF-kB: Nuclear factor kappa B. Statistical differences between the Control and OVA group, $$$: P<0.001. Statistical difference between OVA vs other groups: +: *P*<0.05, ++: *P*<0.01, +++: *P*<0.001. Use the ANOVA test to compare between groups.

Photographs of a lung specimen in different groups (magnification for each group, 10x) (Hematoxylin-eosin staining). A: control group, B: OVA-sensitized group, C: OVA-sensitization and crocin 30 mg/kg, and D: OVA-sensitization and crocin 60 mg/kg. Arrow: destruction of airway epithelium, 1: lung fibrosis, 2: hyperemia, and 3: lymphocyte cell infiltration. Statistical differences between the Control and OVA group, $: *P*<0.05. Statistical difference between OVA vs other groups: +: *P*<0.05. Use the ANOVA test to compare between groups.

## Discussion

The most important findings of the current study are as follows:

1. The OVA-sensitized mice displayed more prominent histopathological changes and increased serum levels of OVA-sensitized Ig-E, which were subsequently modulated by intervention with crocin. 

2. In the OVA-sensitized animals, there was a significant decrease in the expression levels of both Nrf2 and HO-1 mRNA and proteins compared to the control group.

3. IL-17 and NF-kB expression levels were notably higher in the OVA-sensitized group.

4. The administration of crocin in OVA-sensitized mice resulted in a protective impact through the NF-kB and Nrf2/HO-1 pathways.

Asthma is characterized by chronic inflammation of the airways, which is evident through leukocyte infiltration, especially eosinophils (29). Furthermore, findings indicate that patients with asthma display increased Th2 cell activity, leading to the generation of multiple cytokines such as IL-5, IL-4, and IL-13 (3). According to the findings of this study, a significant increase in the accumulation of eosinophils, neutrophils, and lymphocytes in the airways was observed under OVA-sensitization status, with crocin demonstrating preventive effects. These effects were more evident with high concentrations of crocin. The study’s findings revealed that crocin has suppressive effects on inflammatory reactions, aligning with previous research (30). 

With the activation of the inflammatory response in allergic airways and mast cells and bronchial hyperreactivity, it has been observed that an augmented production of IgE occurs, accompanied by the release of different cytokines by eosinophils and mast cells (31). In the current study, it was observed that OVA-sensitized mice had higher amounts of OVA-specific IgE, but the intervention with crocin effectively reduced these levels. It can be inferred that crocin has reduced airway inflammation by decreasing IgE levels, at least partially.

Asthma researchers have directed their attention towards NF-κB, a transcription factor that greatly impacts inflammatory reaction mechanisms. Activating NF-kB leads to the accumulation of inflammatory cells in the airways and their involvement in adaptive immune responses (8). Generally, NF-κB is inactive in the cytoplasm due to binding with IκB. Upon phosphorylation of IκB, NF-κB is released and translocated to the nucleus, which can initiate various inflammatory signaling pathways (32). In allergic airway inflammation cases, NF-kB activation coincides with promoting Th2 and Th1 cytokine expression (32). In this regard, suppressing the NF-κB activation pathway is considered to be one of the essential therapeutic goals for patients with asthma. The results of the current study identified that crocin intervention prevented the augmentation of NF-kB mRNA and protein expression in ovalbumin-sensitized animals. Interestingly, the effects of crocin were observed in a dose-dependent manner. 

In animal studies focused on asthma, it has been discovered that Th17 cells and related cytokines (such as IL-17A) are involved in recruiting inflammatory cells to the airways, especially in cases where asthma is severe (33). Increased expression of IL-17A and IL-17F mRNA and protein has been observed in individuals with obesity-related asthma, suggesting a unique asthma phenotype (4, 34). In addition, Zhang *et al.* revealed that elevated levels of IL-17 were observed in O_3_-induced asthma (35). The results suggest that in severe and refractory asthma, the activation of Th1, Th2, and Th17 cells occurs, leading to the disease’s exacerbation (35). IL-17A has been recognized as a stimulus for neutrophil infiltration in the airways (36). In patients who died from asthma exacerbation, it was found that there were higher numbers of neutrophils than eosinophils (37). Findings from this study revealed a marked increase in IL-17 expression in OVA-sensitized mice relative to the control group. An interesting observation was made when a high dosage of crocin (60 mg/kg) was used, resulting in significant prevention of IL-17 elevation. The findings from this research provide additional evidence that crocin has anti-inflammatory effects in ovalbumin-sensitized animals, as indicated by the reduction of IL-17 expression.

Alongside inflammation, oxidative stress serves as a critical player in the development of asthma (38). Chronic lung diseases like COPD and asthma have been associated with disturbance in the oxidants/anti-oxidants balance (15). It has been observed that asthma status is associated with increased levels of oxidants (such as malonyl dialdehyde (MDA)) and reduced anti-oxidants factors (such as superoxide dismutase (SOD) and glutathione peroxidase (GPx)) (39). Furthermore, researchers have observed a decline in Nrf2 expression, which plays a significant role in maintaining cellular defenses (38). Nrf2 acts as a redox-sensitive transcription factor that becomes activated when exposed to oxidative stress conditions; it then regulates genes and proteins responsible for anti-oxidant and anti-inflammatory defense (40). In Nrf2 null mice, there has been evidence demonstrating the occurrence of exacerbation of AHR, mucus hypersecretion, and eosinophilia – all hallmarks of allergic asthma (41). Under physiological conditions, Nrf2 is in an inactive complex state due to interaction with Kelch-like epichlorohydrin-associated protein 1 (Keap-1) (42). Appropriate extracellular signals lead to the dissociation of Nrf2 from Keap-1 and translocate to the nucleus, interacting with the anti-oxidant-responsive element (ARE) to participate in the transcription of various genes, such as HO-1 (42, 43). HO-1, a part of the heat shock protein family, has anti-oxidant and anti-inflammatory properties (44). Numerous studies have provided evidence indicating that HO-1 exerts interesting effects on asthma by decreasing inflammation, oxidative stress, and the secretion of excess mucus (45). The reduced levels of Nrf2 and HO-1 were evident in the lung tissue of OVA-sensitized mice, in which the intervention with crocin exerted a protective effect. Despite the need for further investigations, it can be inferred from the results that crocin exerted protective effects on animals with asthma, possibly through activation of the Nrf2/HO-1 pathway.

The anti-inflammatory and anti-oxidant effects of saffron and its bioactive compound, crocin, are well-documented in several disorders. Crocin protective effects are thought to be mainly mediated through the modulation of signaling pathways such as NF-kB, T-bet/GATA-3 ratio, miRNAs, signal transducer and transcription activator 6 (STAT6), protein kinase C (PKC), inducible nitric oxide synthase (iNOS), mitogen-activated protein kinases (MAPK/ERK), high-mobility group box 1 (HMGB-1) pathway, endoplasmic reticulum (ER) stress markers, Ca^2+^ /calmodulin-dependent protein kinase 4 (CAMK4), c-JNK, and phosphoinositide-3-kinase (PI3K)/Akt (15-19, 30, 46-48).

One of the suggestions for future studies is to use cell line studies to investigate the possible mechanisms involved in the Nrf2/HO-1 pathway. Demonstrating the influence of crocin in asthma through the NF-κB-Nrf2/HO-1 pathway can be achieved by employing Nrf2 pathway inhibitors or activators as well. On the other hand, the limitation of the current study was that it did not have a positive control group, such as dexamethasone.

## Conclusion

In general, disruption of the Nrf2/HO-1 pathway in asthma is accompanied by airway hyperresponsiveness, neutrophil and eosinophil infiltration, and increased mucus secretion. By reducing Nrf2, it may be possible to observe an increase in NF-κB activity, subsequently leading to the generation of NF-κB-dependent pro-inflammatory cytokines. The crocin intervention reduced airway inflammation, with its effects linked to diminished NF-κB activity and increased expression of Nrf2/HO-1 mRNA and proteins. However, additional studies are needed for a more detailed investigation.

## Data Availability

The data sets used and/or analyzed during the current study are available from the corresponding author upon reasonable request.
